# Supplementary material for: Interaction of 4-ethyl phenyl sulfate with bovine serum albumin: Experimental and molecular docking studies
Source: PLoS One. 2024 Oct 28;19(10):e0309057. doi: 10.1371/journal.pone.0309057 (PMC11516168; doi:10.1371/journal.pone.0309057)
Supplement: S1 File — S1 Table relates to ground state configurations of 4-EPS with every atom’s spatial arrangement: bond length and angle. S2 Table relates to dipole moment, optimized energy Value and RMS gradient normalization value for optimized EPS molecule. (DOCX) [file pone.0309057.s001.docx]

**Supplementary File**

**Interaction of 4-ethyl phenyl sulfate with bovine serum albumin: Experimental and molecular docking studies**

Payal Gulati^a*^, Pratima Solanki^b^, Awadhesh Kumar Verma^b,c^, and Anil Kumar^a^

^a^Gene Regulation Laboratory, National Institute of Immunology, Aruna Asaf Ali Marg, New Delhi - 110067, India

^b^Special Centre for Nanoscience, Jawaharlal Nehru University, New Delhi-110067, India

^c^School of Bioengineering and Biosciences, Lovely Professional University, Phagwara, Punjab - 144001, India

***Corresponding Author: payalgulati@nii.ac.in**


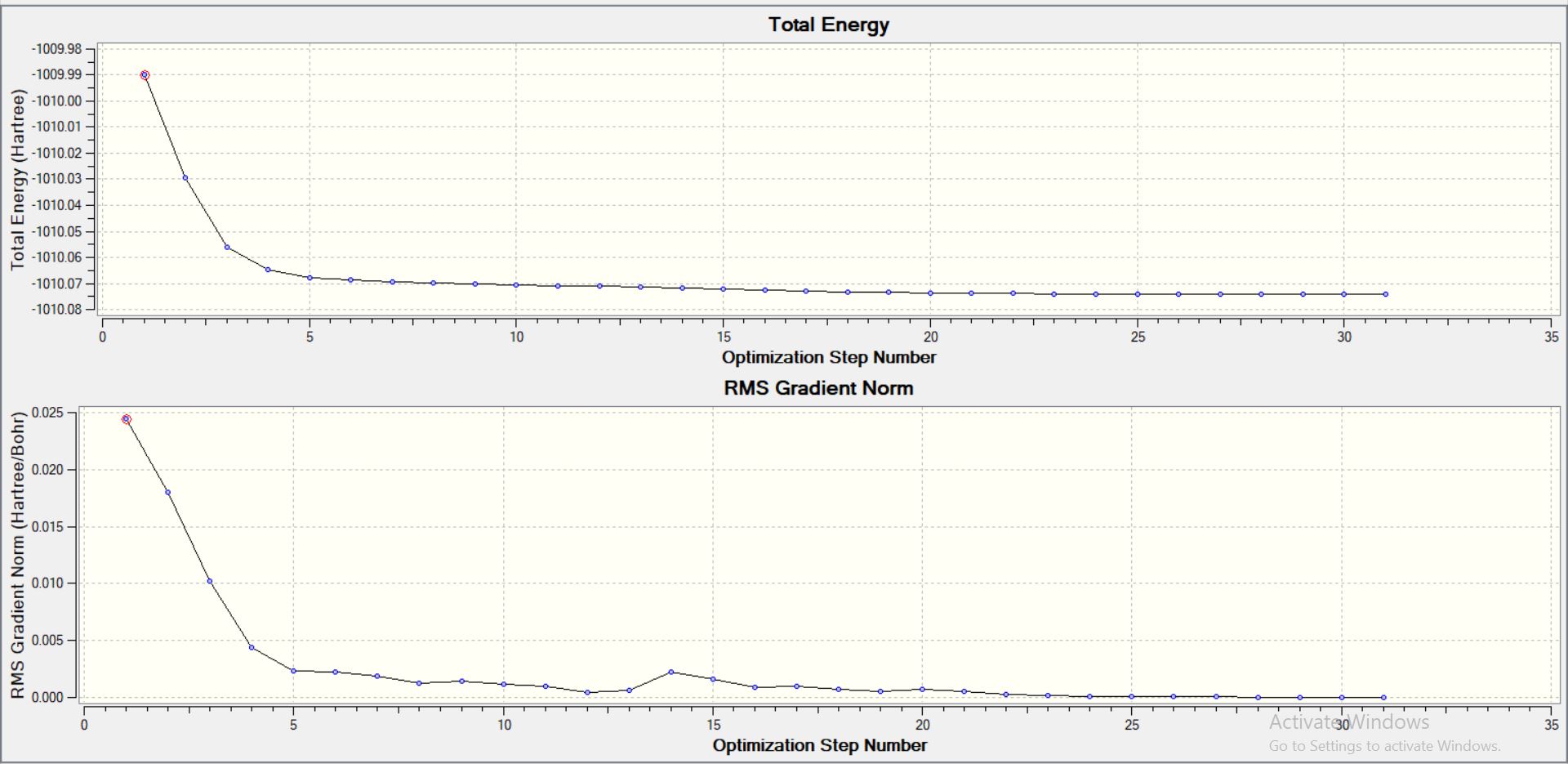


S1_Fig. This is Fig S1 legend. Showing the energy minimization and RMS gradient normalization for 4-EPS with optimization stem number.

S1_Table. This is the Table S1 Title. Ground state configurations of 4-EPS with every atom’s spatial arrangement: bond length and angle.

| **4-EPS** | | | | | |
| --- | --- | --- | --- | --- | --- |
| **Bond Length (Å)** | **Unoptimised** | **Optimised** | **Bond Angle (°)** | **Unoptimised** | **Optimised** |
| S=O (2) | 1.46960 | 1.46960 | O=S-O (3) | 109.47122 | 109.47121 |
| S-O (2) | 1.67000 | 1.67000 | S=O-H (1) | 109.47122 | 109.47120 |
| O-H (1) | 0.96000 | 0.96000 | C=C-H (4) | 120.00000 | 119.99995 |
| C-C (5) | 1.54000 | 1.54000 | C-C-H (3) ring | 120.00000 | 120.00003 |
| C-H (9) | 1.07 | 1.07 | C-C-H (5) | 109.47122 | 109.47124 |
| C=C(3) |  |  | H-C-H (3) | 109.47122 | 109.47122 |

S2_Table. This is the Table S2 Title. Dipole moment, Optimized energy Value and RMS gradient normalization value for optimized EPS molecule.

| **Dipole Moment** | **0.6472 Debye** |
| --- | --- |
| **Energy Value** | **-1009.99 a.u.** |
| **RMS Gradient Norm** | **0.02450 a.u.** |
